# Influence of Vanadium 4+ and 5+ Ions on the Differentiation and Activation of Human Osteoclasts

**DOI:** 10.1155/2017/9439036

**Published:** 2017-08-29

**Authors:** Matthias A. König, Oliver P. Gautschi, Hans-Peter Simmen, Luis Filgueira, Dieter Cadosch

**Affiliations:** ^1^Department of Traumatology, University Hospital Zurich, Zurich, Switzerland; ^2^Département de Neurosciences Cliniques, Geneva University Hospital, Geneva, Switzerland; ^3^School of Anatomy and Human Biology, University of Western Australia, Perth, WA, Australia; ^4^Department of General and Trauma Surgery, Triemlispital, Zurich, Switzerland

## Abstract

**Background:**

In the pathophysiology of implant failure, metal ions and inflammation-driven osteoclasts (OC) play a crucial role. The aim of this study was to investigate whether vanadium (V) ions induce differentiation of monocytic OC precursors into osteoresorptive multinucleated cells. In addition, the influence of V ions on the activation and function of* in vitro* generated OC was observed.

**Methods:**

Human monocytes and osteoclasts were isolated from peripheral blood monocytic cells (PBMCs). Exposition with increasing concentrations (0–3 *μ*M) of V4^+^/V5^+^ ions for 7 days followed. Assessment of OC differentiation, cell viability, and resorptional ability was performed by standard colorimetric cell viability assay 3-(4,5-dimethylthiazol-2-yl)-5-(3-carboxymethoxyphenyl)-2-(4-sulfophenil)-2H-tetrazolium (MTS), tartrate-resistant acid phosphatase (TRAP) expression, and functional resorption assays on bone slides during a period of 21 days.

**Results:**

No significant differences were noted between V4^+^/V5^+^ ions (*p* > 0.05). MTS showed significant reduction in cellular viability by V concentrations above 3 *μ*M (*p* < 0.05). V concentrations above 0.5 *μ*M showed negative effects on OC activation/differentiation. Higher V concentrations showed negative effects on resorptive function (all *p* < 0.05) without affecting cell viability. V4^+^/V5^+^ concentrations below 3 *μ*M have negative effects on OC differentiation/function without affecting cell survival.

**Conclusion:**

Vanadium-containing implants may reduce implant failure rate by influencing osteoclast activity at the bone-implant interface. V-ligand complexes might offer new treatment options by accumulating in the bone.

## 1. Introduction

Metal-based implants have become essential and very successful treatment tools in dental and orthopedic trauma surgery. Implant failure rates are usually well below 5% for the first two years. The rates may increase up to 10–20% under local inflammatory conditions, including periodontitis and allergic reactions against the metal implant [1]. To date, surgical revision is the gold standard in treating implant failure. With an increasingly ageing population, implant failure with the consequent need of revision surgery will have a clinical and economic significance in the future.

Aseptic loosening (AL) is the leading cause of failure of total joint arthroplasty [2]. Aside from the well investigated and recognized role of wear debris (in the nanometer range) in the initiation and development of AL, over the last years, there has been increasing evidence that involvement of metal ions released by biocorrosion might influence AL by enhancing osteolysis and decreasing osseointegration [3, 4].

Various metal alloys used in metal implants contain vanadium (V), which gives the alloy favorable physicochemical and mechanical properties. Several* in vitro* and* in vivo* studies have investigated the bioactivity of V and different V compounds. It has been demonstrated that V compounds have an insulin and growth factor mimicking action [5, 6]. In addition, V compounds largely influence phosphatases, a group of enzymes mainly associated with the cell membrane [7]. Phosphatases, specifically the tartrate-resistant acid phosphatase (TRAP), play a paramount role in osteoclastic activities. Inhibiting TRAP might reduce the risk of AL.* In vivo* studies carried out to investigate the biodistribution of V compounds showed that V accumulates predominantly in the bone, kidney, spleen, and liver after 24 h of administration [8]. A recently published study has also shown that V compounds are able to regulate osteoblastic growth [9]. In particular, V ions released by biocorrosion from metal implants and accumulated in the bone could exert specific effects on bone turnover and more specifically on OC. Little is known about the potential biological effects of V ions on OC. In this study, we investigate the hypothesis that V4^+^ and V5^+^ ions may interfere with OC differentiation and activation using a well-established* in vitro* human OC model, as well as an* in vitro* bone resorption model.

## 2. Methods

### 2.1. Isolation of Peripheral Blood Monocytic Cells and Generation of Osteoclasts

The study protocol established by Lionetto et al. was used in the experiments [10]. Ficoll-gradient centrifugation (Amersham Biosciences, Uppsala, Sweden) was used to isolate pooled peripheral blood monocytic cells (PBMCs) from buffy coats of healthy blood donors. RPMI-1640 plus GlutaMAX™ medium (RPMI) (Gibco/Invitrogen, Auckland, New Zealand) supplemented with 5% human serum and 1% antibiotics (10,000 units/mL penicillin G sodium, 10,000 *μ*g/mL streptomycin sulfate, and 25 *μ*g/mL amphotericin B (Gibco standard medium)) was the culture medium for PBMCs at 37°C (humified, 5% CO_2_, in 25 cm^2^ tissue culture flasks (Sarstedt)). The nonadherent PBMCs were discarded and the adherent PBMCs were washed twice using 0.1 M phosphate-buffered saline (PBS, pH = 7.2), scraped off, and resuspended in standard medium after 1 h of culturing. These cells were used as monocytic cells (MCs) as described below.

To generate the OC, cell cultures of adherent PBMCs were supplemented with OC differentiation cytokines (10 ng/mL recombinant human macrophage-colony stimulating factor (hM-CSF) and 10 ng/mL recombinant human receptor activator of NF-*κ*B ligand (hRANKL) (ReproTech, Rocky Hill, NJ, USA)).

### 2.2. Cell Culture Conditions

The MC, MC supplemented with OC differentiation cytokines, and mature OC were subsequently exposed to increasing concentrations (0 to 3 *μ*M) of bis(maltolato)oxovanadium(IV) (BMOV) V4^+^ or oxidized bis(maltolato)oxovanadium(V) (oxBMOV) V5^+^ ions for 7 days. Cell viability was assessed using 3-(4,5-dimethylthiazol-2-yl)-5-(3-carboxymethoxyphenyl)-2-(4-sulfophenyl)-2H-tetrazolium (MTS) colorimetric assay (Promega, Madison, WI, USA). The assays were repeated four times for each experimental condition, and the mean optical light density was recorded using a microplate reader (Labsystems, Helsinki, Finland) at an absorbance of 492 nm.

### 2.3. Detection of TRAP Using ELF97 and Flow Cytometry

The endogenous tartrate-resistant acid phosphatase (TRAP) activity was detected by using the phosphatase substrate ELF97 (Molecular Probes, Eugene, OR, USA) as described previously [11], to assess OC differentiation in the presence of the different concentrations of V4^+^ or V5^+^ ions. Cell fixation was performed after 7 days, using a culture medium containing 1% paraformaldehyde. After being washed twice with distilled water, the fixed cells were subsequently incubated with 200 *μ*M ELF97 in a 110 mM acetate buffer staining solution for a period of 20 min. Cells were characterized for expression of surface markers by using fluorescence-labeled mouse monoclonal antibodies binding specifically to human HLA-DR (phycoerythrin (PE) fluorescent labeled; Becton Dickinson Biosciences, San Jose, CA, USA) and CD45 (PerCP fluorescent labeled; Becton Dickinson Biosciences). To account for the background signal and autofluorescence, isotype antibodies and unstained controls were used.

A FACS Vantage Cell Sorter with a UV laser was used for sample analysis. Approximately 10^5^ gated cells were analyzed. The ELF97 was excited at 350 nm, and the TRAP-related signal was collected using a 530/30 band pass filter (515–545 nm). The PE and PerCP fluorochromes were excited using a 488 nm laser. The signal was collected using a 575/26 and a 675/20 band pass filter. The data was further analyzed using the Flowjo v8.5.3 software package (Treestar, Ashland, OR, USA).

### 2.4. Osteoclast Resorptive Function on the Dentine Slices

Evidence of OC function and bone resorptive activity was assessed using lacunar resorption assays on the dentine slices, as previously described [3]. Whale dentine slices (diameter 15 mm, thickness 0.5 mm) were cut from a sperm whale tooth, purchased from Kaempf (Osborne Park, Western Australia, collected prior to 1972, before the ban on whale hunting in Australia). Adherent cells (4 × 10^5^ cells/well) were cultured on dentine slices. The slices were washed with water and left overnight in a 0.25% ammonium hydroxide solution after a culturing period of 21 days to remove all cellular material. Staining was performed with 1% toluidine blue dye in 1% sodium borate for 10 min. A Nikon Inverted Microscope Eclipse TE 300 (Nikon Instruments, Melville, NY, USA) and a Nikon CCD digital camera were used for documentation. The whole surface of the slice was examined. The DXM 1200F-ACT-1 image processing software (Nikon) was used to analyze the images. A dark-blue excavation on the dentine surface with a clear rim of unchanged and unstained original surface located between neighboring pits was defined as a resorption pit. The extent of resorption was determined from the number of pits formed and by calculating the total planar surface area of the resorption pits. The diameter of 30 pits/slice was measured and the mean value used to calculate the surface of the number of pits counted. The number of pits was counted using a single blinded observer.

### 2.5. Calculations and Statistical Analysis

The data was analyzed using the SPSS for Windows software package (v15.0; SPSS Inc., Chicago, IL). Independent sample* t*-tests were conducted to determine whether the mean ion concentrations were significantly greater than the minimum detection limits. A one-way ANOVA test was used to test the differences in the mean in the expression of the TRAP, as well as differences in the mean number of resorption pits and the surface area across the various cell cultures. A *p* value of *p* < 0.05 was considered to be statistically significant.

## 3. Results

### 3.1. Cell Viability

**Figure 1:**
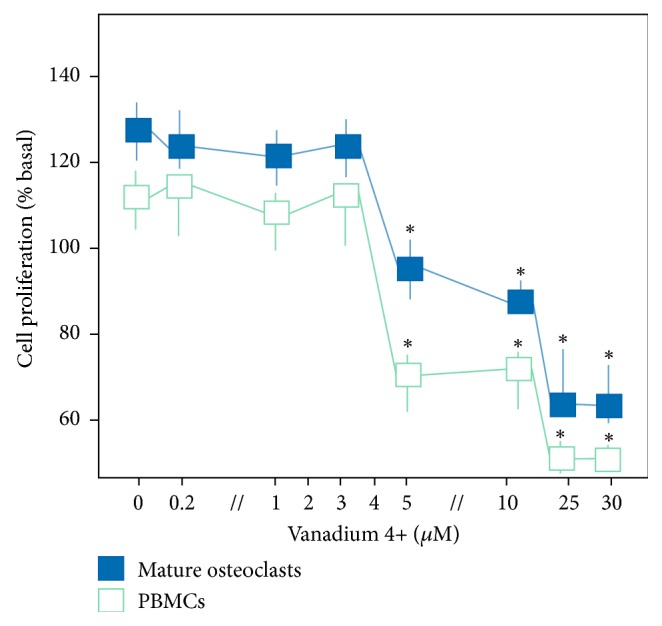
Assessment of cell viability. Cell proliferation (percentage of basal proliferation) of monocytic cells and osteoclasts under increasing concentration of vanadium 4^+^ ions. No differences were noted in the same concentrations of vanadium 5^+^ ions (*p* > 0.05). ^*∗*^*p* < 0.001.

Nontoxic concentrations of V4^+^ and V5^+^ ions that did not greatly decrease the MC and OC viability were defined by MTS colorimetric assays. A significant reduction in MC and* in vitro* generated OC was seen at concentrations of V4^+^ or V5^+^ ions greater than 3 *μ*M. No significant change in MC and OC viability was found when using concentrations up to 3 *μ*M, indicating the toxic concentrations for MC and OC (Figure 1). Toxicity of V4+ and V5+ concentrations above 3 *μ*M was additionally confirmed by Annexin-5 staining. No significant difference was found between V4^+^ and V5^+^ ions (data not shown) (*p* > 0.05). Subsequently, increasing concentrations (0 to 3 *μ*M of V4^+^ or V5^+^) were defined as standard conditions for the experiments.

### 3.2. Detection of TRAP Using ELF97 and Flow Cytometry (FACS)

**Figure 2:**
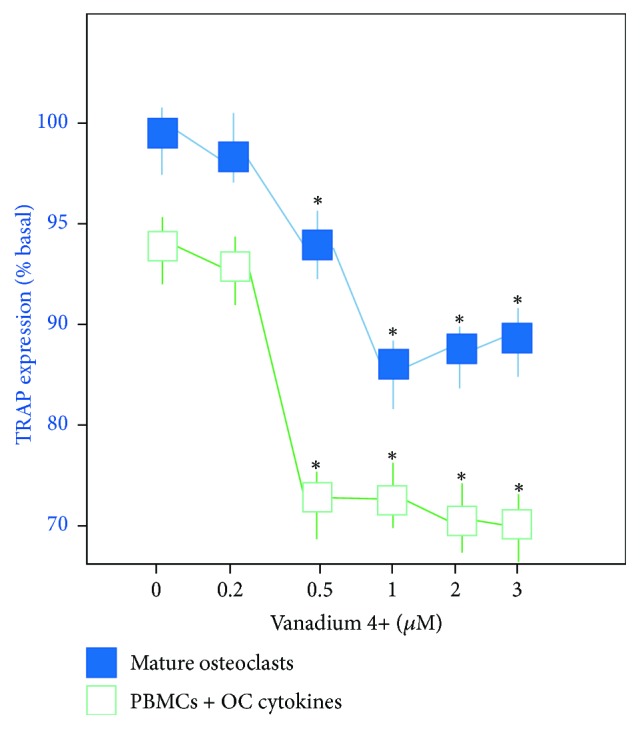
Quantitative analysis of TRAP expression by osteoclasts and monocytes supplemented with osteoclast differentiation cytokines in the presence of increasing concentrations (0 to 3 *μ*M) of V4^+^ ions. Mean fluorescence intensity (MFI) indicates the average TRAP expression per cell. The MFI value indicates relative TRAP expression when compared with the control (MFI = 121). No differences were noted between the same concentrations of V4^+^ and V5^+^ ions (*p* > 0.05) (^*∗*^*p* < 0.05).

All OC cultures were TRAP-positive while all the MC cultures supplemented with OC differentiation cytokines showed a significantly reduced expression of TRAP, indicating OC differentiation inhibition in the presence of V4^+^ or V5^+^ for all the concentrations (Figure 2). All cultures of MC exposed only to V4^+^ or V5^+^ (no supplementation with OC differentiation cytokines) showed no expression of TRAP. Quantitative FACS analysis showed that TRAP expression was significantly decreased in the OC cultures exposed to increasing concentrations of V4^+^ or V5^+^ ions compared with OC cultured without any V ions (*p* < 0.05) (Figure 2).

### 3.3. Qualitative and Quantitative Assessment of Osteoclastic Resorptive Function on the Dentine Slices

The resorptive function was assessed on the dentin slides after the discovery that V4^+^ and V5^+^ ions had an essential effect on OC differentiation for the selected concentrations.

**Figure 3:**
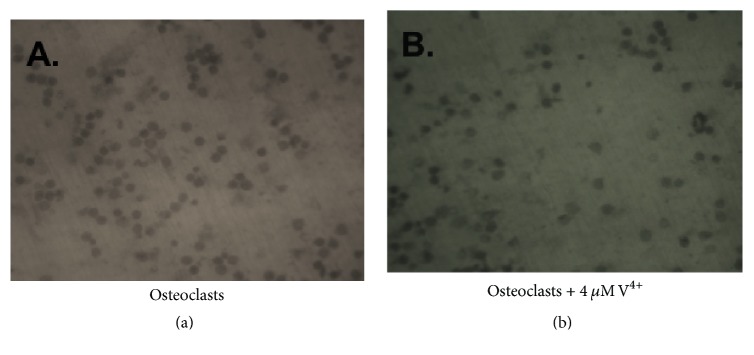
Assessment of bone resorption. The images show a representative example of resorption pits (dark spots) on dentine slides after 21 days of incubation under different culture conditions. (a) Untreated osteoclasts. (b) Osteoclasts + 4 *μ*M vanadium 4^+^ ions.

**Figure 4:**
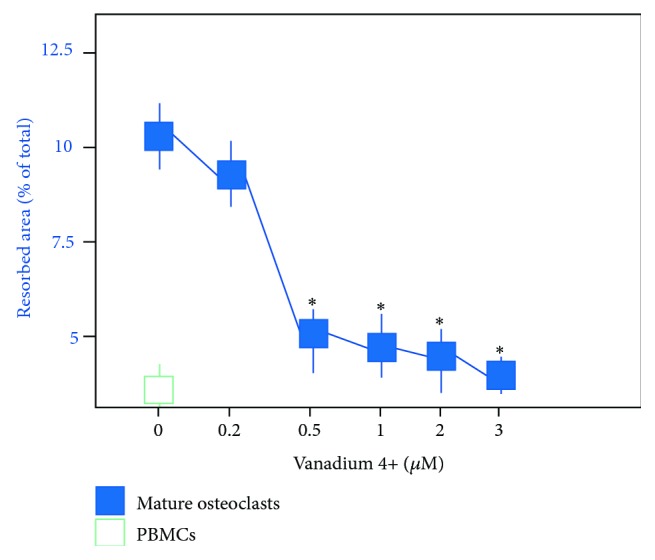
Histogram representing the mean area (percentage of total area ± SE) of resorption on dentine slides after 21 days in culture under different conditions. Note the significant decrease of dentin resorption by osteoclasts exposed to V4^+^ ions. No differences were noted between the same concentrations of V4^+^ and V5^+^ ions (*p* > 0.05) (^*∗*^*p* > 0.05).

OC were cultured with and without V4^+^ or V5^+^ ions on dentin slides for a period of 21 days. The extent of lacunar resorption was analyzed (Figure 3). Between the different conditions, qualitative and quantitative differences in the patterns of absorption were seen. Compared to the control samples, exposition to increased V4+ or V5+ ions led to smaller sized resorption pits, while the resorption pits of untreated OC were larger. Osteoclasts incubated with the different concentrations of V4^+^ or V5^+^ ions showed a significantly decreased number of resorption pits and resorption area of the total dentine surface compared to untreated OC (Figure 4). Monocytes, as a negative control, showed only very few resorption pits (22 ± 3.9 pits/mm^2^) with small dentine area resorption (0.28%, Figure 4). Untreated OC showed an average of 801 ± 13.2 resorption pits/mm^2^ with a dentine resorption area of 10.19% (*p* < 0.001). Regarding the resorption area, no difference was detected between V4^+^ and V5^+^ ions (*p* > 0.05). Resorption features were not seen on the dentine slices incubated without cells.

## 4. Discussion

The hypothesis that V4^+^ and V5^+^ ions are able to interfere with OC differentiation and bone resorptive activity was clearly supported in this study. The hypothesis was based on previous data showing the strong effects of different metal ions on bone metabolism as well as the tendency of V ions/compounds to accumulate in bone cells [3, 4, 8].

The integrity of the skeleton as well as the osseous integration of intercortical metal implants (e.g., total joint arthroplasty) requires the regulated activity of bone-forming cells (osteoblasts) and bone-resorbing cells (OC). Numerous growth factors and cytokines with pro- and antiapoptotic effects are produced in the bone, regulating the balance between osteoformation and resorption [9, 11, 12]. Thus, imbalanced bone cell activity results in severe bone alterations and tissue loss leading in the worst cases to implant loosening without osseointegration. The term “osseointegration” is defined as incorporation of nonvital components in a predictive and reliable way into vital bone. This anchorage mechanism is specifically important if cementless implants are used [13, 14]. In addition, altered TRAP levels in bone pathologies like osteoporosis might compromise osseointegration [15].

Metal ions have been shown to affect bone turnover through a variety of both direct and indirect mechanisms. It has been already demonstrated that Ti4^+^ ions act directly on bone cells by enhancing OC differentiation [3]. In line with this, Co2^+^ and Cr3^+^ ions affected human osteoblasts by inhibiting the release of alkaline phosphatase, enhancing apoptosis, RANKL, and osteoprotegerin (OPG) expression, with enhanced osteoclastogenesis and osteolysis [16, 17]. Furthermore, metal ions induce elevated proinflammatory cytokine secretion such as interleukin- (IL-) 1*α*/*β*, IL-6, and tumor necrosis factor- (TNF-) *α* [4, 18]. TNF-*α* has a direct influence on OC precursors, whereas IL-6 and IL-1*α*/*β* act indirectly by increasing osteoblast RANKL and M-CSF expression, which directly drives osteoclastogenesis [4]. Several studies have reported the bioactivity of vanadate, vanadyl, and different V4^+^ complexes on bone metabolism [19–22]. Low concentrations (<25 *μ*M) of V4^+^ compounds influence the proliferation and differentiation of osteoblast-like cells in culture, while higher concentrations have inhibitory and cytotoxic effects on osteoblasts. V compounds stimulate the protein tyrosine phosphorylation through the inhibition of protein tyrosine phosphatases (PTPases) [23]. The presented results confirm the cytotoxicity of high concentrations (>3 *μ*M) of V4^+^ and V5^+^ ions on MC and OC in culture. This study demonstrates the inhibitory action of V4^+^ and V5^+^ ions at concentration < 3 *μ*M on OC differentiation and activity* in vitro*. This is based on several observations: (a) significantly decreased expression of TRAP in cultures of MC supplemented with OC differentiation cytokines and exposed to V4^+^ or V5^+^ ions, (b) significantly decreased TRAP expression in cell cultures of mature and functional OC exposed to V4^+^ or V5^+^ ions, and (c) significantly reduced bone resorption by OC exposed to V4^+^ or V5^+^.

## 5. Conclusion

V-containing implants may reduce OC activity at the bone-implant interface and thereby decrease bone resorption and ultimately the rate of implant failure. Primary osseointegration is supported by suppressing local TRAP activity. Additionally, V complex development with different ligands might be an alternative strategy of use in skeletal tissue engineering especially when considering their tendency to accumulate in bone.

## Abbreviations

AL: Aseptic loosening
ARCBS: Australian Red Cross Blood Service
Co: Cobalt
Cr: Chrome
MC: Monocytic cells
MTS: 3-(4,5-Dimethylthiazol-2-yl)-5-(3-carboxymethoxyphenyl)-2-(4-sulfophenil)-2H-tetrazolium
*μ*M: Micromol
nm: Nanometer
OC: Osteoclasts
PBMC: Peripheral blood monocytic cell
PE: Phycoerythrin
TRAP: Tartrate-resistant acid phosphatase
V: Vanadium
BMOV: Bis(maltolato)oxovanadium(IV)
oxBMOV: Oxidized bis(maltolato)oxovanadium(V).
